# Annotations

**Published:** 1897-12-18

**Authors:** 


### Annotations.

### Charity Mendicants.

It is with great satisfaction that we record the fact that the street collections organised in connection with the Hospital Saturday Fund are to be discontinued, and we congratulate Mr. Acland and the Council upon their courageous insistence on these reforms. We have long protested against street collections, for we have seen that the discredit they brought upon the Fund was in no way compensated by the money they produced. As a fact, the money coming in from this source has been steadily diminishing. It is not, however, on account of their failure to bring in money that we think them so injurious; it is not merely because the custom of begging for money in the public streets for a reputable object like the Saturday Fund has led to a host of imitators begging for all sorts of objects, some of which were devoid of all guarantee of their genuineness; nor is it even that the system of turning girls out into the streets to rattle collecting-boxes and to beg from every passer-by is degrading in fact and bad in tendency; these things are each of them sufficient to condemn the practice, but beyond them all is the patent fact that this begging from the well-to-do, not for direct contributions to the hospitals, but for help towards a fund which is ostensibly a fund contributed by working men, is a public affront to the working community, and is sure to alienate them from the object in hand. There is no reason why the working people of London, who after all are the people for whose benefit the hospitals of London are primarily maintained, should not make regular collection among themselves, in their own workshops and through their own organisations, and present every year, as a free gift to the hospitals from which they receive such manifold advantages, a noble sum, a sum of which they as a class might be justly proud, a sum gathered out of their own pockets, representing sacrifice and self-denial, as truly a charitable gift for the relief of the suffering around them as the guineas and the cheques by which these institutions are mainly supported. But how can they do these things; how can they ask their fellows to join in getting up this fund; how can they take a pride in this fund when it has been got together, while, so far as the public knows, it is collected in the streets by a form of pertinacious importunity which would not be allowed for a moment except under the cloak of charity from people of another class altogether? It was a system which was bad in itself, and was an excuse for much that was worse; and we hope that now that the Hospital Saturday Fund has given it up the police will clear the London streets of all "charity mendicants."

### Isolation Nurses for District Work.

It used to be a common complaint against isolation hospitals that the staff kept regularly in them was so small that when an epidemic came it had to be reinforced by nurses who had little or no training, and often were not of the best character. In many hospitals this fault has now been remedied, and the staff is large enough for any probable emergency. But, as there are times when there is comparatively little infectious disease, it follows that for part—occasionally a considerable part—of every year these nurses have little to do. They may be employed in making and mending linen, and in similar tasks; but even so this leaves the most valuable part of their capacities lying fallow. A suggestion made by a medical officer of health would, if carried out, do much to remove the reproach of keeping nurses in idleness, and would at the same time lessen that terror of the isolation hospital which is common among the poor. Why should not the nurses belonging to the hospital act as district nurses during the time that work is slack? The number of preventable deaths, especially of children, among the poor, is enormous. Ignorance of diet, neglect of small ailments, unconscious carelessness of every kind, lead to fatal results. If the hospital nurses could visit outside, many poor mothers would, through their visits, get lessons in hygiene, which would prevent their committing the errors that played havoc with the health of the patient to whom the nurse was first sent. Another benefit, less direct but not less important, would come from making the people acquainted with the nurse. The poor have a deep distrust of the fever hospital. The ruses they will try to prevent a patient being carried off to the hospital would be amusing if the consequences were not so serious. We have heard even of the patient being hidden under the bed while the sanitary officials were in the house, and were being solemnly assured that there was no fever there. Now, if the nurses of the fever hospital became, as by this means they might, the familiar friends of the poor in the neighbourhood, the dread of the hospital which causes these deceptions would largely disappear. They would be entrusting those they love to friends, whose kindness and skill they had already tested. Even now the habit of sending a nurse with the ambulance waggon makes the removal easier to many an anxious mother, who is comforted by the sight of the kindly face. But how much more would this be the case if the nurse were one whom the mother already knew.

### Vestries and Public Health.

Some people hold firmly to the belief that many things which are now undertaken by private enterprise would be better done by public bodies. Theoretically this is no doubt right enough, but in practice we have to take our vestries and other petty rulers as we find them, and until they show some little improvement in their proceedings we can hardly feel encouraged in handing over to them increased power and authority. Every vestry is the sanitary authority for its own district, appointed by Parliament to carry out the provisions of the Public Health Act. Yet the Mansion House Council on the Dwellings of the Poor finds plenty to do in keeping these same sanitary authorities up to their work. The recent friction between the Council and the Whitechapel Board of Works is but one of the many examples which are constantly occurring in which, but for the intervention of an independent body, the powers that be would remain content to fold their hands. The occurrence of diphtheria year after year in the same house may surely be taken as calling for something more than an ordinary inspection of its sanitary condition, and we fail to see why any Vestry should object to the intervention of a body like the Mansion House Council to ensure to the dwellers in such a house that proper protection from insanitary conditions which is their legal right. When the Mansion House Council finds itself short of work we shall begin to have a little more faith than we can confess to at the present time in the efficiency of our vestries as guardians of the public health.

